# Reliability Volume: a novel metric for surgical skill evaluation

**DOI:** 10.3389/fmed.2025.1591043

**Published:** 2025-09-17

**Authors:** Zhipu Yu, Qinghua Liu, Jin Zhang

**Affiliations:** ^1^School of Physics and Electronic Information, Yan'an University, Yan'an, China; ^2^Yan'an University Affiliated Hospital, Yan'an, China

**Keywords:** surgical skill, repetitive training, trajectory similarity, reliability, fatigue

## Abstract

This study introduces Reliability Volume (RV), an integrated metric combining trajectory similarity with empirical reliability estimation using threshold counts to evaluate surgical skill during repetitive training. RV quantifies both spatial precision and the probability of consistent task execution, addressing limitations of single-session metrics that neglect fatigue and performance drift. Applied to knot-tying with assistive devices, RV jointly reflects spatial accuracy and performance consistency over multiple sessions. Our results show that RV reliably tracks learning progression and is readily compatible with real-time (closed-loop) feedback systems, providing a dynamic, comprehensive, and practice-oriented assessment framework.

## 1 Introduction

Surgical skill evaluation methods can be broadly categorized as subjective and objective. Subjective evaluations, including expert ratings and self-assessments, remain prevalent yet suffer from rater bias, inconsistent standards, and inefficiency (1, 2). Objective evaluations quantify surgical gestures, eye movements, or instrument trajectories (3–7), but often require specialized hardware, complex analyses, and substantial expertise, limiting practicality (8). In pursuit of more accurate assessments, quantitative metrics such as force-based (9), time-based (4, 10), and spatial indicators (e.g., path length and smoothness) (11, 12) have received considerable attention. Methods including Dynamic Time Warping (DTW), Hidden Markov Models (HMM), and kinematic feature extraction are widely used to evaluate the quality and similarity of surgical movements (13–15). Recent reviews also highlight the rapid growth of computer vision and AI for objective skill assessment and training across open, laparoscopic, and robotic platforms (16, 17).

Repetitive practice of fundamental skills is particularly important given limited operating room opportunities, duty-hour restrictions, and ethical constraints. Although repetition can improve accuracy, efficiency, and trainee confidence, most evaluation metrics focus on single sessions and do not adequately account for cumulative fatigue and performance drift during repetitive training.

Fatigue is a key external factor. Kahol et al. reported cognitive deterioration due to fatigue and sleep deprivation in virtual reality simulations that was not captured by operative time alone (18). More recent syntheses show mixed but concerning effects of surgeon fatigue on performance and outcomes and call for direct, within-task measures rather than retrospective proxies (19).

**Figure 1:**
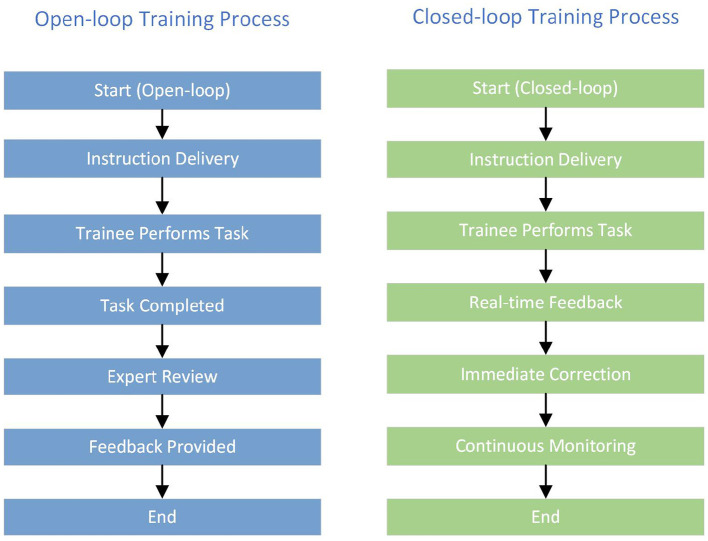
Comparison of open-loop and closed-loop training processes.

To contrast single-session (open-loop) and repeated-session (closed-loop) training, we compare traditional methods with real-time feedback systems, as illustrated in Figure 1 (20–23). Open-loop approaches provide delayed feedback only after task completion, limiting opportunities for in-task correction. Closed-loop approaches deliver immediate feedback and continuous monitoring, enabling trainees to adjust actions promptly and mitigate negative effects from fatigue and other external factors.

Addressing these limitations, we propose *Reliability Volume* (RV), derived from Euclidean distance (13), working volume (11), and empirical reliability estimation (24, 25). Unlike traditional metrics, RV quantifies a trainee's consistency in real-time, closed-loop environments by jointly capturing short-term spatial accuracy and long-term consistency. RV thus provides a comprehensive, realistic, and practical framework that bridges theoretical modeling and real-world training.

## 2 Reliability volume and its calculation

**Figure 2:**
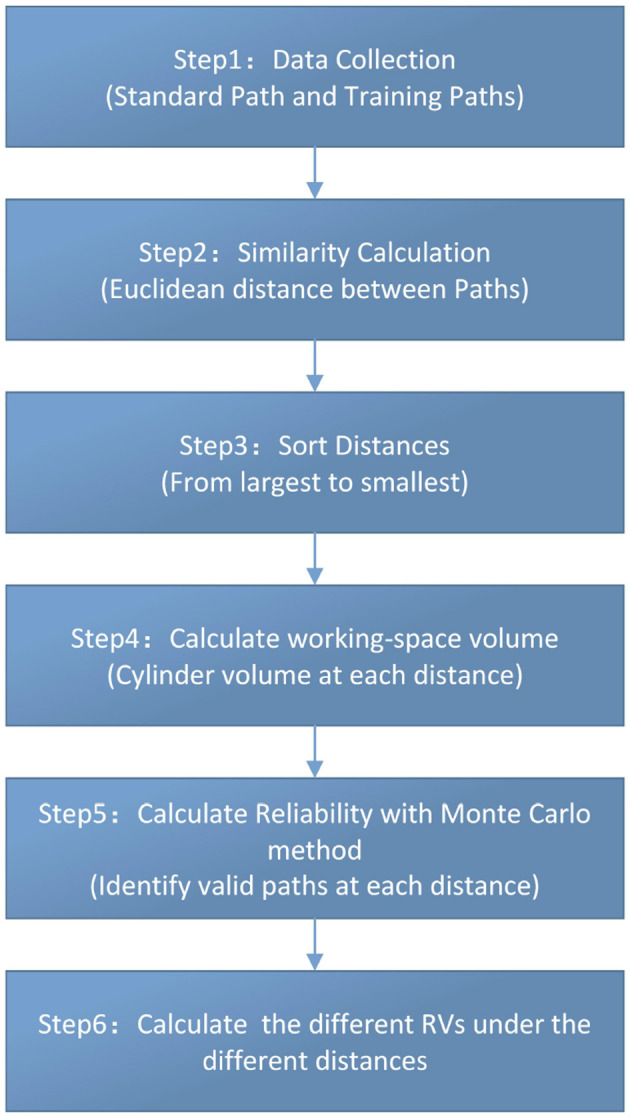
Calculation flowchart.

RV is a bivariate descriptor reported as an ordered pair (*R, V*), where *R* is the probability of successfully completing the task within specified conditions, and *V* represents the corresponding working-space volume. A lower *R* indicates a higher probability of failure; a smaller *V* indicates closer alignment with the standard path. The workflow is shown in Figure 2.


(1) $$\begin{array}{l} \text{RV}=\left(R,V\right). \end{array}$$

Specifically, the calculation steps are as follows.

**Step 1: capture standard and training paths**

We define two sets of 3D trajectories:

- **Standard path** *S* = {*s*_1_, *s*_2_, …, *s*_*N*_}, with $s_{n}=\left(x_{n}^{\prime },y_{n}^{\prime },z_{n}^{\prime }\right)$.
- **Training path** *T*_*m*_ = {*t*_*m*1_, *t*_*m*2_, …, *t*_*mN*_} for the *m*-th repetition, where *t*_*mn*_ = (*x*_*mn*_, *y*_*mn*_, *z*_*mn*_), *m* ∈ {1, …, *M*}.

**Step 2: pointwise euclidean deviation**

The deviation at index *n* of repetition *m* is


(2) $$d_{mn}=\|t_{mn}-s_{n}\|=\sqrt{{\left(x_{mn}-x_{n}^{\prime }\right)}^{2}+{\left(y_{mn}-y_{n}^{\prime }\right)}^{2}+{\left(z_{mn}-z_{n}^{\prime }\right)}^{2}}.$$

**Step 3: order the deviations**

Collect all *d*_*mn*_ and sort in descending order to obtain *D*_*s*_ = {*d*_max_, …, *d*_*j*_, …, *d*_min_}, where *d*_*j*_ denotes a distance (radius) threshold. (Here *D*_*s*_ denotes the multiset of all *d*_*mn*_).

**Step 4: working-space volume**

**Figure 3:**
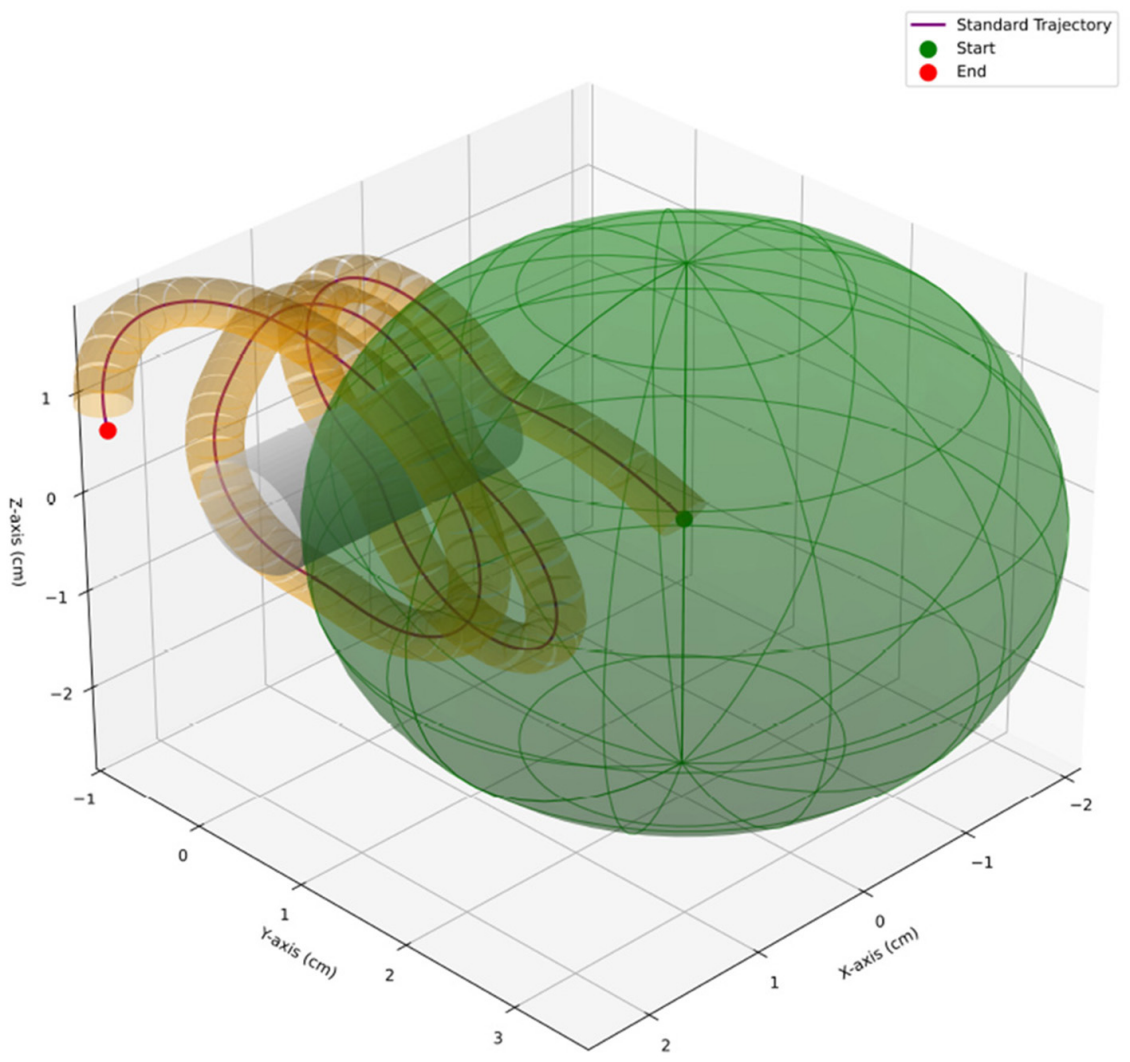
Working-space volume (cylindrical tube around the standard path) vs. conventional working volume.

Unlike the conventional *working volume* defined as a sphere whose radius equals the average distance from a hand-centered point (11), we model a *working-space volume* as a cylindrical tube coaxial with the standard path (Figure 3). For a given threshold *d*_*j*_, the working-space volume is


(3) $$\begin{array}{l} V_{j}=\pi d_{j}^{2}h \end{array}$$

where *h* is the arc length of the standard path and *d*_*j*_ is the tube radius.

**Step 5: empirical reliability estimation**

The Monte Carlo method is a powerful statistical tool for evaluating the ability to complete a specified surgical task within a given time and environment (24, 25). Therefore, for each distance *d*_*j*_, the state function is *Z* = *g*(*m, n*) = *d*_*mn*_ − *d*_*j*_. Based on this state equation, *d*_*mn*_ − *d*_*j*_ = 0 can divide the variable space into a failure space and a reliability space, and the working-space volume, a cylinder centered on the target path, defines the reliable space. Moreover, count the number of paths *n*_*j*_, which *d*_*mi*_ does not exceed *d*_*j*_, and compute reliability *R*_*j*_ as:


(4) $$\begin{array}{l} R_{j}=\frac{n_{j}}{M} \end{array}$$

where *R*_*j*_ is the reliability corresponding to distance *d*_*j*_.

**Step 6: Reliability Volume**

Finally, the Reliability Volume at threshold *d*_*j*_ is


(5) $$\begin{array}{l} {\text{RV}}_{j}=\left(R_{j},V_{j}\right). \end{array}$$

## 3 Experiment: knot-tying with assistive devices

### 3.1 Path data collection

Path data were collected using an optical motion-tracking system (Beijing DuLiang Technology Co.) to monitor hand movements during the experiment. The core hardware and software configurations of this system are detailed in Table 1.

**Table 1:** Optical Motion-tracking system core configuration for path data collection.

**Component type**	**Equipment name**	**Brand and model**	**Key technical specifications**
Core Hardware	Optical Motion-Tracking Camera	NOKOV Mars1.3H	Resolution: 1,280 × 1,024 (1.3 million pixels); Max acquisition frequency (full resolution): 240 Hz (adjustable); Power supply: Power over ethernet (POE); Interface: GigE/POE
	8-port POE switch (power supply)	NOKOV POE8/8-ONV1	POE power ports: 8; Data transmission port: 1; Total power output: 128 W
Core Software	Motion Tracking & Data Analysis Software	NOKOV XINGYING	Data processing: FPGA edge computing; Compatibility: Supports Windows/Linux/MATLAB/Simulink/ROS

**Figure 4:**
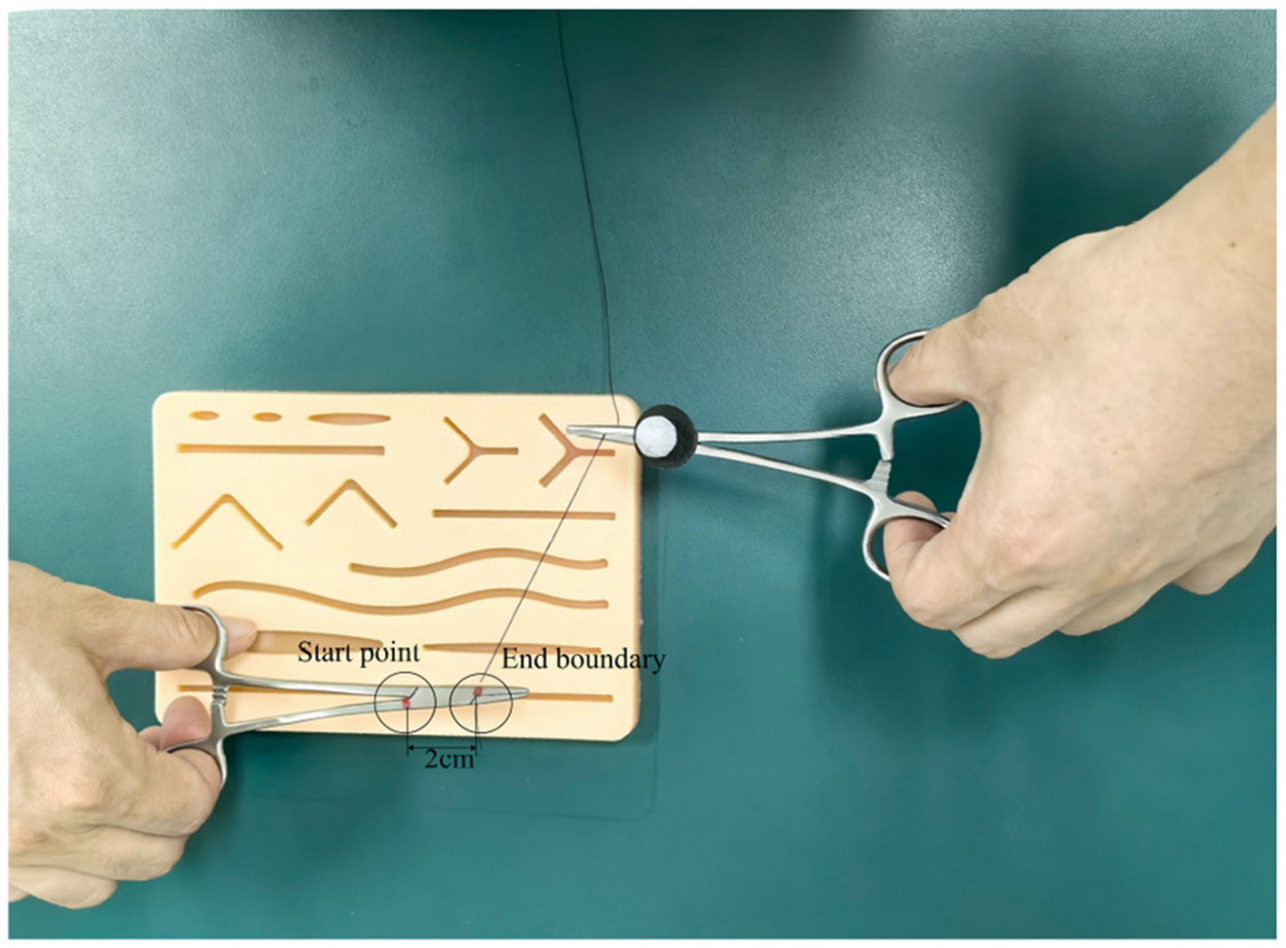
Working environment.

**Figure 5:**
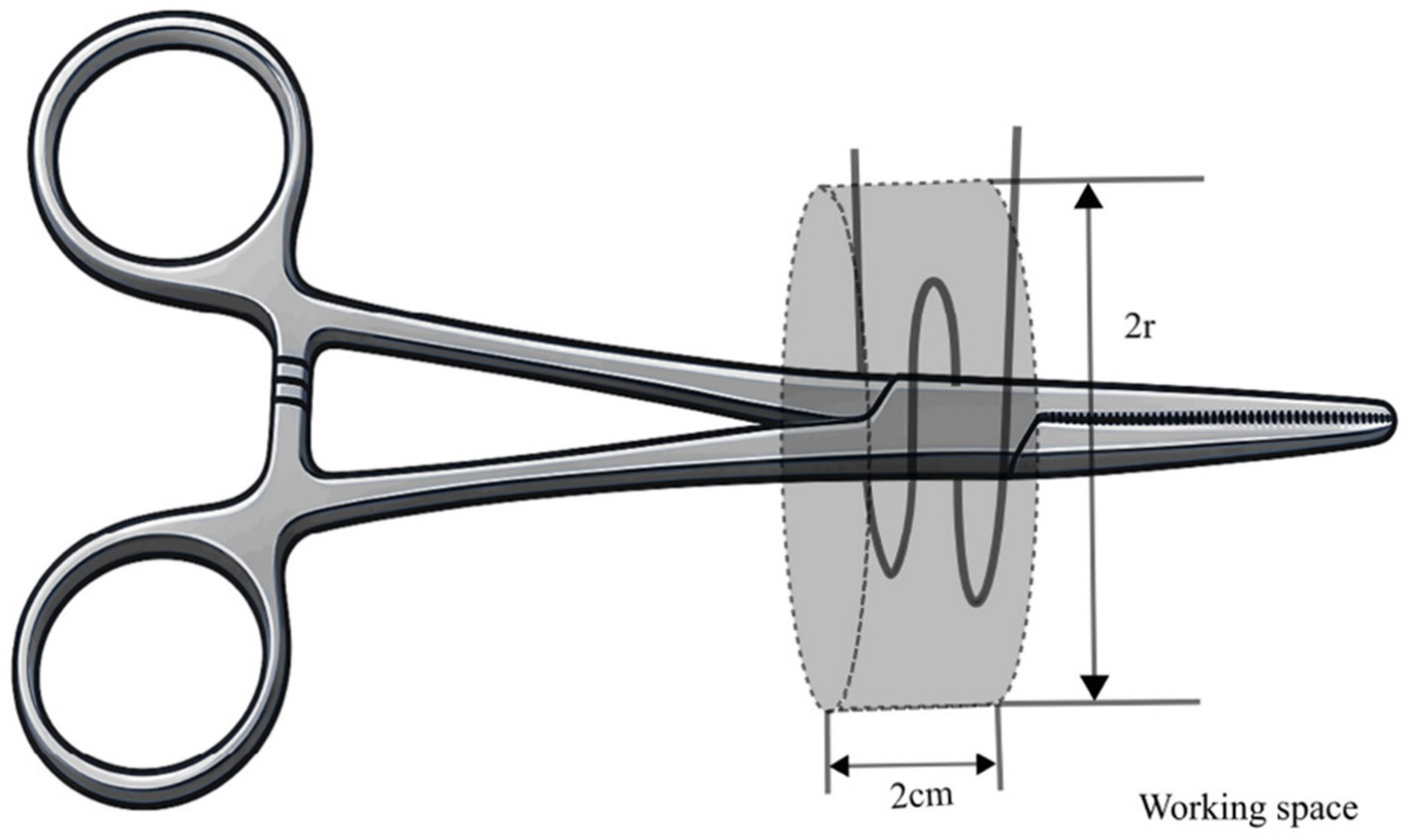
Schematic of the working-space volume.

As shown in Figure 4, a 12 mm reflective marker was affixed to a pair of hemostatic forceps. Trainees used the instrument to tie a suture around a needle holder, completing two full loops at a self-selected comfortable speed. Each repetition started at a prescribed start point and ended at a predefined boundary. The task was performed within a cylindrical workspace (Figure 5) with a fixed height of 2 cm and variable radius *r* (cm).

All trajectories were recorded at a uniform sampling frequency and saved in CSV format to ensure consistent path length for subsequent computational comparisons.

### 3.2 Participants

Participants included four students, three surgical residents, two attending surgeons, and one associate chief surgeon. The associate chief surgeon performed the knot-tying procedure once to define the standard path. Each trainee then imitated the task 50 times at a self-selected comfortable speed, with no time limit imposed. A total of nine trainees (five male, four female) participated, with demographic information indicated in the captions of Figures 7–15. Path data were collected via the motion-tracking system.

### 3.3 Standard path

**Figure 6:**
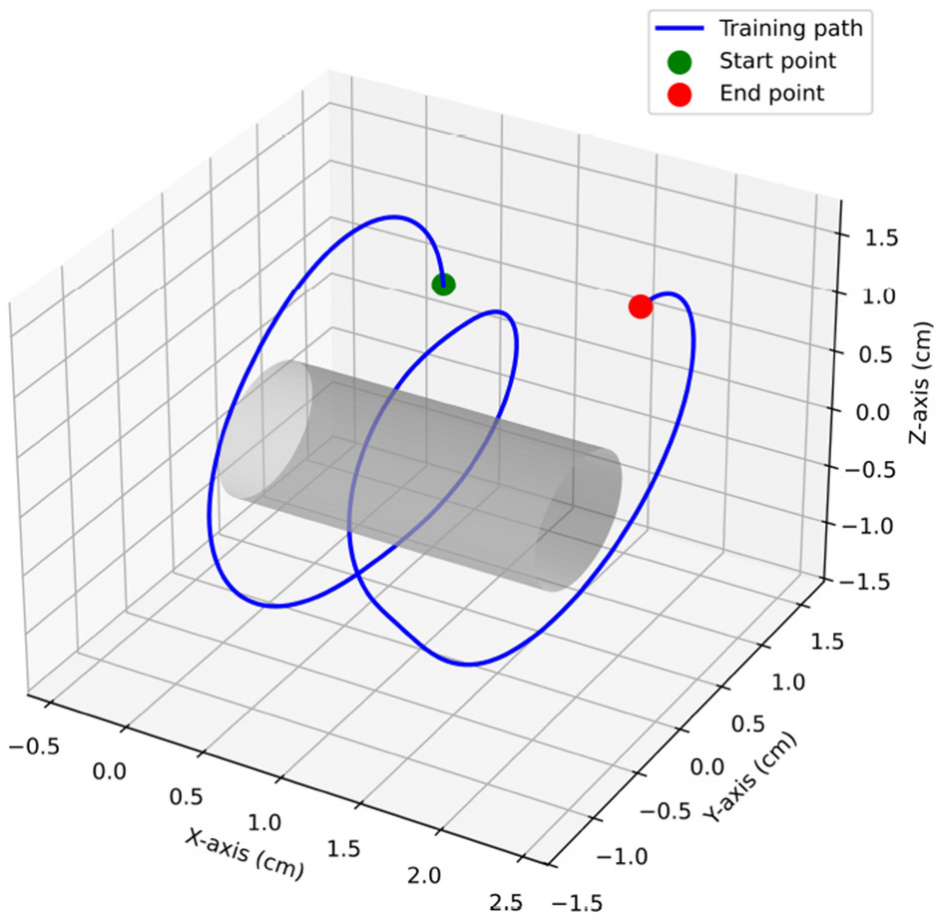
Standard path.

Figure 6 presents the standard path generated by the associate chief surgeon, which served as the reference for trainees.

### 3.4 Reliability Volume (RV) results

**Figure 7:**
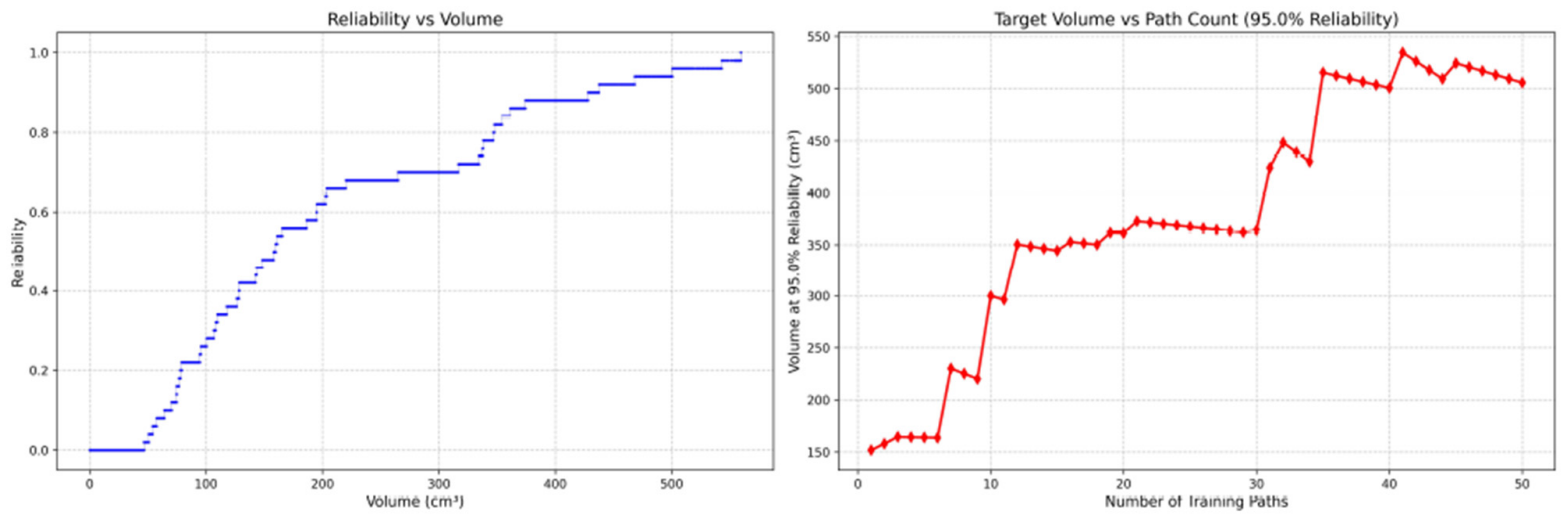
Reliability Volume (RV) results for student 1 (male). **(Left)** *R* vs. *V*; **(Right)** *V* vs. repetitions at *R* = 0.95.

**Figure 8:**
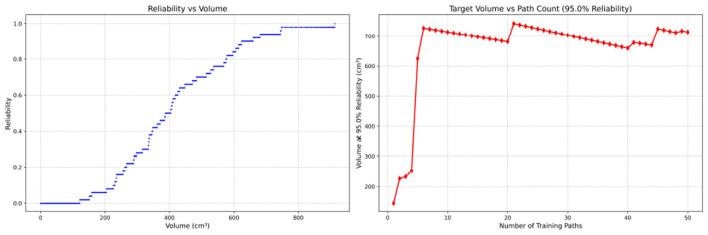
Reliability Volume (RV) results for student 2 (male). **(Left)** *R* vs. *V*; **(Right)** *V* vs. repetitions at *R* = 0.95.

**Figure 9:**
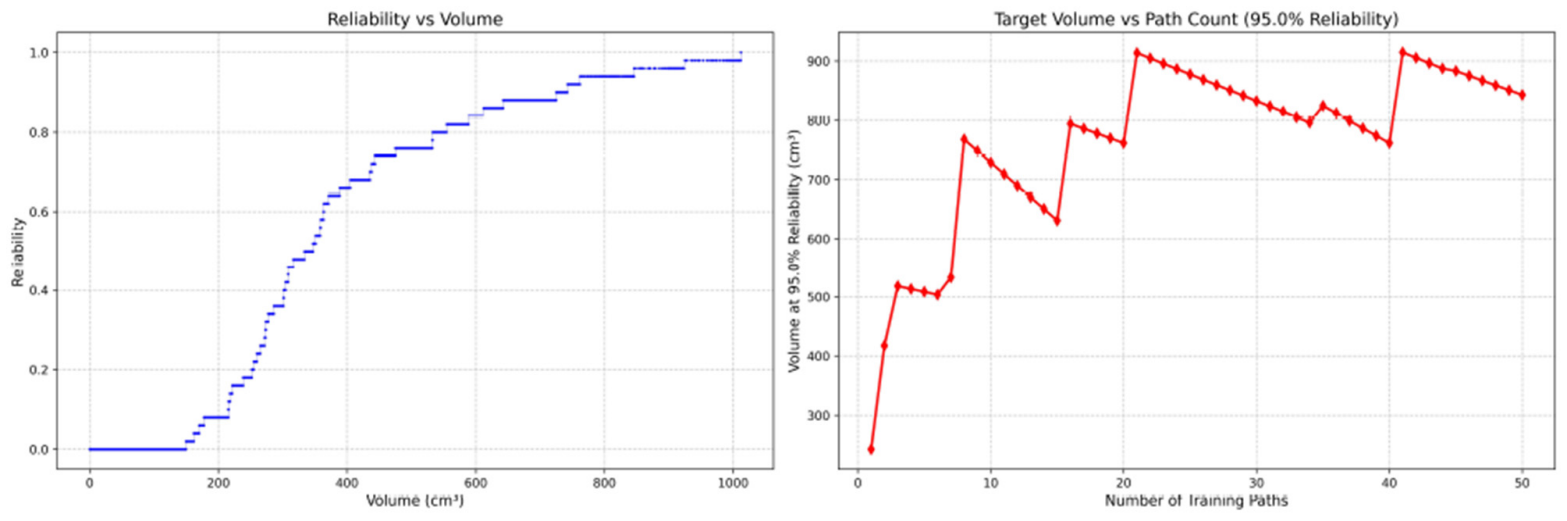
Reliability Volume (RV) results for student 3 (male). **(Left)** *R* vs. *V*; **(Right)** *V* vs. repetitions at *R* = 0.95.

**Figure 10:**
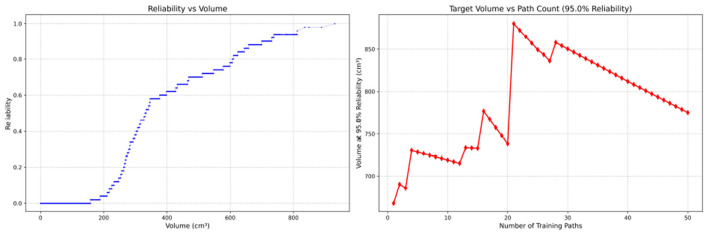
Reliability Volume (RV) results for student 4 (female). **(Left)** *R* vs. *V*; **(Right)** *V* vs. repetitions at *R* = 0.95.

**Figure 11:**
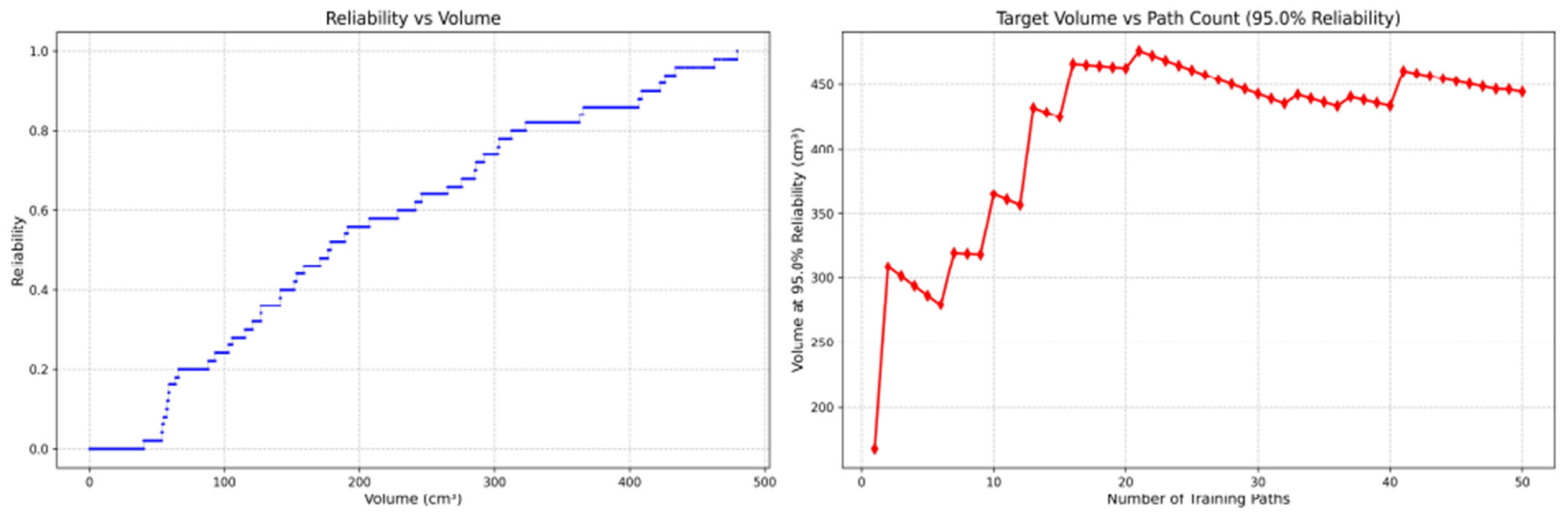
Reliability Volume (RV) results for surgical resident 1 (male). **(Left)** *R* vs. *V*; **(Right)** *V* vs. repetitions at *R* = 0.95.

**Figure 12:**
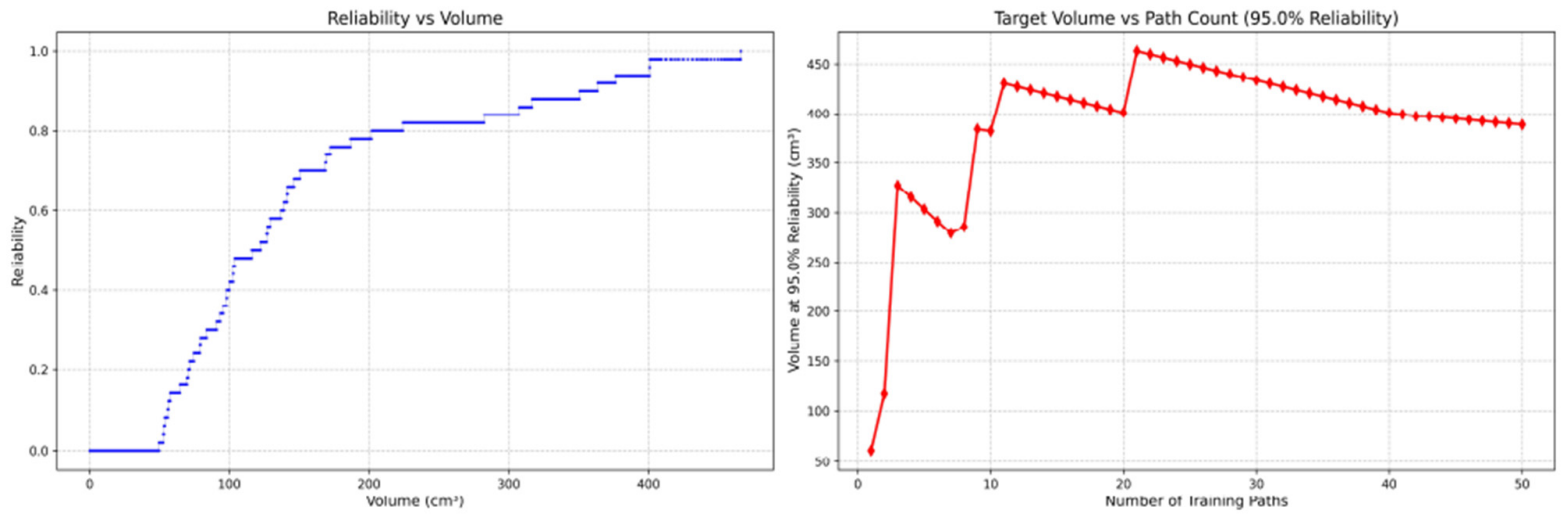
Reliability Volume (RV) results for surgical resident 2 (female). **(Left)** *R* vs. *V*; **(Right)** *V* vs. repetitions at *R* = 0.95.

**Figure 13:**
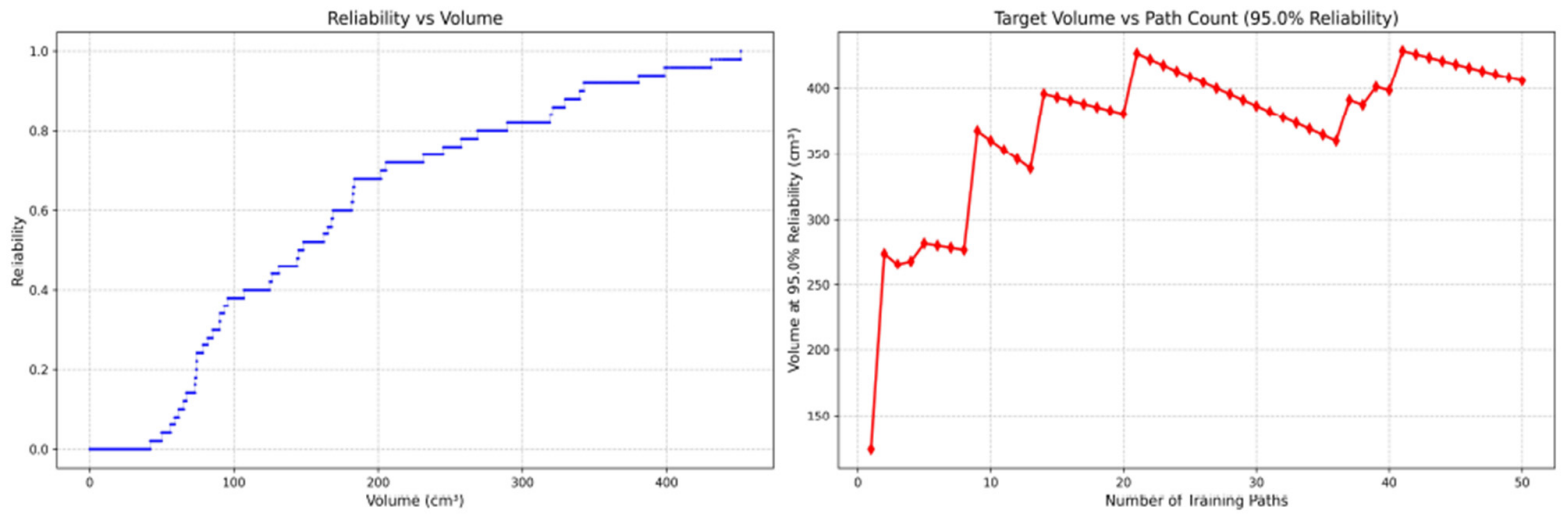
Reliability Volume (RV) results for surgical resident 3 (female). **(Left)** *R* vs. *V*; **(Right)** *V* vs. repetitions at *R* = 0.95.

**Figure 14:**
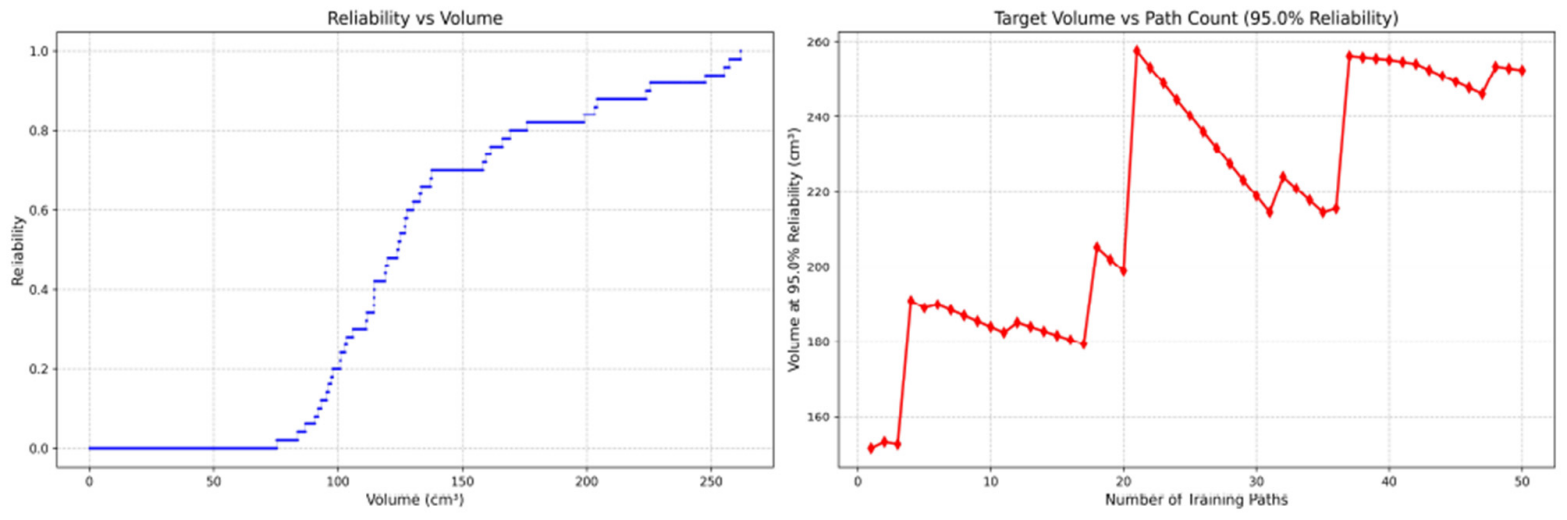
Reliability Volume (RV) results for attending surgeon 1 (female). **(Left)** *R* vs. *V*; **(Right)** *V* vs. repetitions at *R* = 0.95.

**Figure 15:**
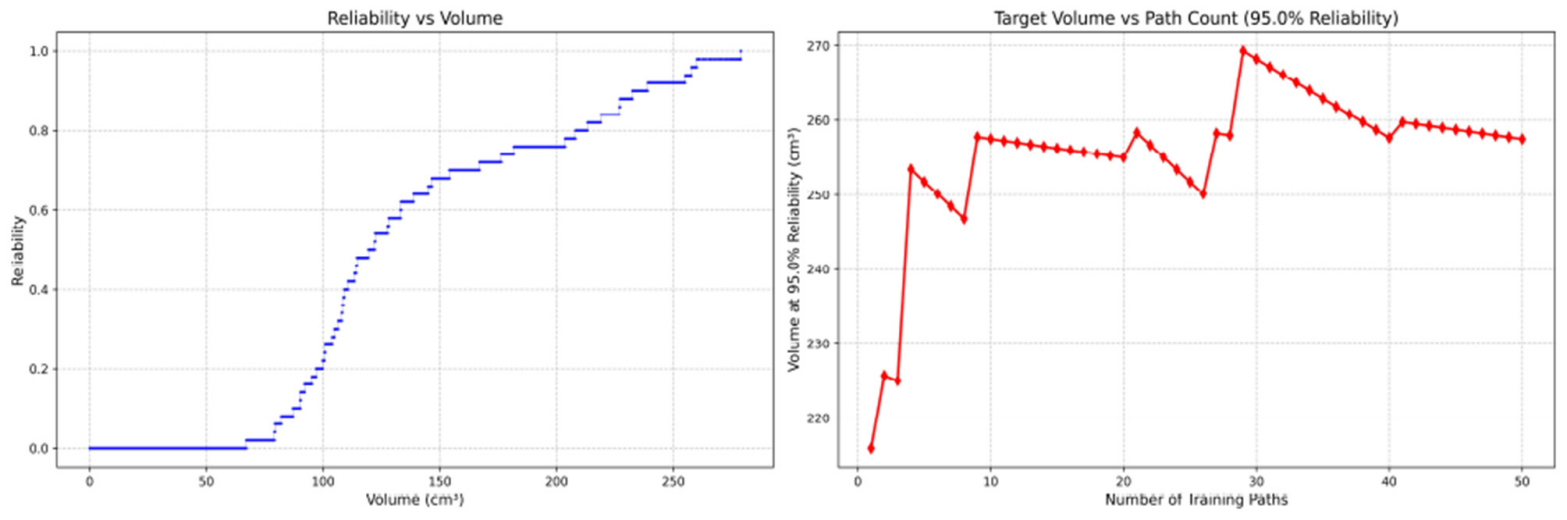
Reliability Volume (RV) results for attending surgeon 2 (male). **(Left)** *R* vs. *V*; **(Right)** *V* vs. repetitions at *R* = 0.95.

Since only the horizontal displacement between the start and end points was constrained—with training paths also being influenced by trainees' experience and physical condition—the actual training paths diverge from the standard path. Accordingly, the Reliability Volume (RV) results are grouped by role: Figures 7–10 (students), Figures 11–13 (surgical residents), and Figures 14, 15 (attending surgeons). In each figure, the left panel shows how *R* varies with the working-space volume *V* at *M* = 50 repetitions; the right panel shows how the working-space volume *V* varies with the number of repetitions when *R* = 0.95.

In the first panels, students generally operate at smaller working-space volumes *V* (i.e., higher spatial precision relative to the standard path) but exhibit broader transitions in reliability from *R* ≈ 0 to *R* ≈ 1, indicating greater performance variability compared with experienced participants. This observation is consistent with previous reports that experienced operators tend to emphasize stability, whereas novices often trade stability for precision (11, 12).

In the second panels, RV reveals training dynamics that are often obscured by traditional single-metric summaries. When fixing *R* = 0.95, a favorable trend is a reduction in *V* with increasing repetitions, reflecting improved precision at a constant success probability. For example, student2, student4, and resident 2 show extended intervals of negative correlation between repetition count and *V*, suggesting more effective practice results.

By contrast, some participants demonstrate positive correlations or non-monotonic patterns. For instance, RV snapshots for student1 at the 10th, 25th, and 50th repetitions (Table 2) reveal that reliability at a fixed volume (e.g., 427.84 cm^3^) can fluctuate (0.90 → 0.96 → 0.90). Similarly, for student2, RV snapshots at the same repetitions (Table 3) demonstrate variability at a constant volume (e.g., 613.33 cm^3^), with reliability shifting from 0.90 → 0.84 → 0.88.

**Table 2:** Reliability Volume (RV) calculations for student 1 at different repetitions.

**10 repetitions**		**25 repetitions**		**50 repetitions**	
**Reliability**	**Volume (cm^3^)**	**Reliability**	**Volume (cm^3^)**	**Reliability**	**Volume (cm^3^)**
0.90	427.84	0.96	427.84	0.90	427.84
0.90	427.26	0.96	427.26	0.90	427.26
0.80	427.18	0.92	427.18	0.88	427.18
0.80	427.12	0.92	427.12	0.88	427.12

**Table 3:** Reliability Volume (RV) calculations for student 2 at different repetitions.

**10 repetitions**		**25 repetitions**		**50 repetitions**	
**Reliability**	**Volume (cm^3^)**	**Reliability**	**Volume (cm^3^)**	**Reliability**	**Volume (cm^3^)**
0.90	613.33	0.84	613.33	0.88	613.33
0.90	613.17	0.84	613.17	0.88	613.17
0.80	613.07	0.80	613.07	0.86	613.07
0.80	613.02	0.80	613.02	0.86	613.02

Clearly, this indicates that a one-size-fits-all imitation training approach may not be suitable for all trainees. While some individuals can achieve improved precision at a high success probability after completing 50 repetitive training sessions, others may not demonstrate such progress.

### 3.5 Fatigue and dynamic feedback

In terms of fatigue, the ability to complete a specified surgical task under defined conditions is closely linked to fatigue accumulation with increasing repetitions. RV offers a practical means to *capture* such effects: fluctuations in *R* at a fixed *V* across repetitions are consistent with transient fatigue or distraction.

For training management, we propose a simple stopping rule compatible with closed-loop feedback: define a reliability change threshold (e.g., |Δ*R*| ≥ 0.05) at a fixed volume. When within-session reliability changes by at least this amount, the session should be paused and skill evaluated using the last stable RV point (the measurement immediately preceding the change). For example, at a working-space volume of 427.84 cm^3^, student1 should stop imitation training at the 10th repetition, as reliability declined from 0.96 to 0.90. At 613.07 cm^3^, student2 should stop at the 25th repetition, as reliability shifted from 0.80 to 0.86. Notably, this stopping rule should account for gradual changes, and this will be explored in future research.

Thus, RV is not only an integrated metric for quantifying task success probability under specified conditions, but also a dynamic measure that reflects fluctuations caused by fatigue or distraction.

## 4 Discussion

### 4.1 Practicality of RV for capturing skill development

As with conventional working volume (11), RV reflects the expected gradient of spatial economy with increasing experience. In our data, the maximum of working-space volume (at *R* = 1) decreased consistently across groups: students ( ≈ 853.57 cm^3^), surgical residents (≈465.62 cm^3^), and attending surgeons (≈270.33 cm^3^). Similarly, the average working volume was 164.50 cm^3^ for students, 66.52 cm^3^ for residents, and 18.30 cm^3^ for attending surgeons.

**Figure 16:**
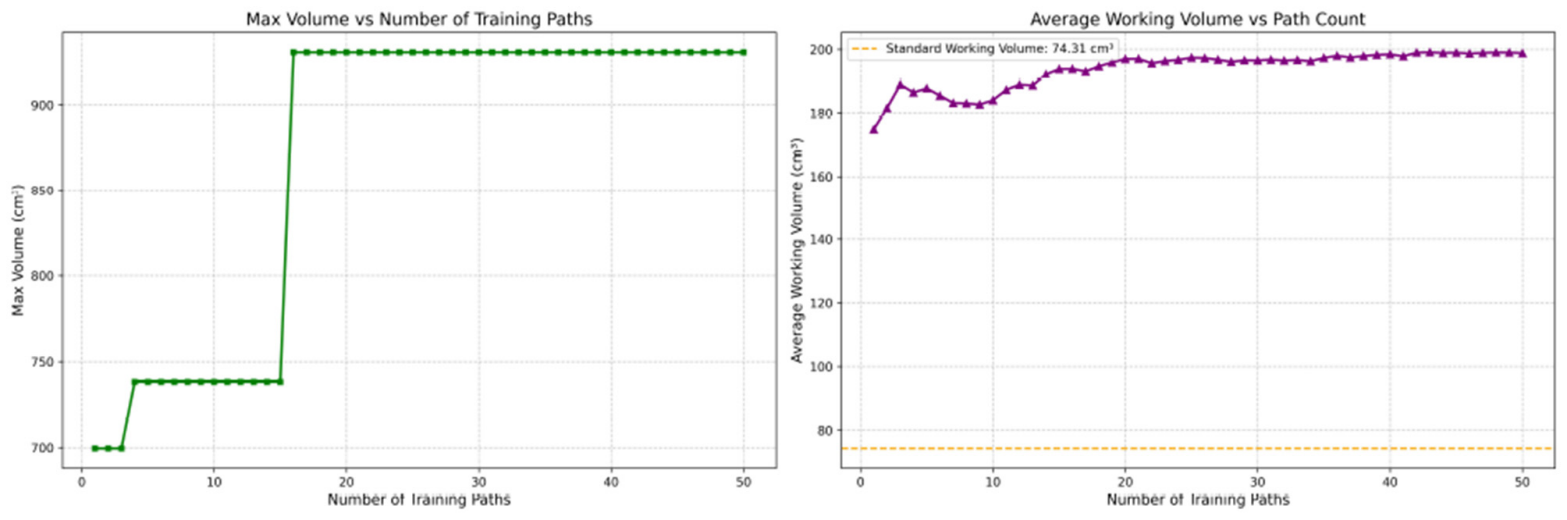
Comparison of Reliability Volume (RV) and conventional working volumes for student 2.

Figure 16 and Table 4 highlight why RV-derived volumes may diverge from conventional working volume. The RV tube radius is defined by the worst-case deviation from the standard path (maximal *d*_*mn*_), whereas the conventional working volume relies on the average distance from a hand-centered point. When fatigue or other uncertainties cause occasional large deviations, the RV maximum volume remains anchored to its tolerance definition and is comparatively stable. By contrast, the average-based working volume is more sensitive to fluctuations.

**Table 4:** Comparison of RV and conventional working volumes for student 2.

**Number of training paths**	**Max RV volume (cm^3^)**	**Working volume (cm^3^)**
10	738.23	196.13
20	930.56	216.59
30	930.56	195.13
40	930.56	204.63
50	930.56	190.08

Thus, although both RV and conventional working volume can stratify experience, RV provides greater practical utility by integrating *all* repetitions within a closed-loop framework (Figure 3). Compared with established metrics such as path length, smoothness, and working volume, RV emphasizes consistency across repetitions rather than single-session snapshots, thereby offering complementary information for comprehensive skill assessment.

### 4.2 Perceived value and implications

Currently, Reliability Volume (RV) primarily focuses on spatial consistency; however, incorporating task duration represents a critical future extension, as prolonged execution may also serve as an indicator of skill variability. While this study demonstrates the feasibility and practicality of the RV metric, the potential impacts of fatigue and other confounding factors require further investigation. Notably, moderating variables such as gender and prior health status were not included in the current analysis. Future research should therefore enroll larger and more diverse cohorts, integrate direct fatigue assessments, and evaluate additional clinical tasks. Furthermore, given that the number of repetitions was used as a proxy for actual training time in this study, integrating RV into automated real-time feedback systems could enhance training efficiency and skill retention by delivering immediate, actionable guidance (16, 17).

## 5 Conclusion

We propose Reliability Volume (RV), an integrated metric that combines trajectory similarity with an empirical reliability-based framework to assess surgical skill in repetitive, realistic training settings. RV quantifies both spatial precision and the probability of consistent task execution, addressing limitations of single-session metrics that overlook fatigue and performance drift. Evidence from knot-tying tasks demonstrates that RV effectively captures consistency over repetitions and reveals trade-offs between precision and reliability. Future work will broaden participant diversity, evaluate additional training scenarios, and investigate the integration of RV into automated real-time feedback systems.

## Data Availability

The raw data supporting the conclusions of this article will be made available by the authors, without undue reservation.
